# 3,3′-[Biphenyl-4,4′-diylbis(­oxy)]diphthalic acid

**DOI:** 10.1107/S1600536811053219

**Published:** 2011-12-21

**Authors:** Ningning Zhao, Wenjun Li, Zhidong Chang, Changyan Sun, Hongxia Fan

**Affiliations:** a Department of Chemistry and Chemical Engineering, University of Science and Technology Beijing, Beijing 100083, People’s Republic of China

## Abstract

In the title mol­ecule, C_28_H_18_O_10_, the two central benzene rings form a dihedral angle of 31.0 (1)°. In the phthalic acid fragments, the carb­oxy groups in the *meta* positions are approximately coplanar with the attached benzene rings, being inclined to their planes at 2.7 (1) and 10.3 (1)°, while the carb­oxy groups in the *ortho* positions are twisted from the benzene ring planes by 83.5 (1) and 75.4 (1)°. In the crystal, O—H⋯O hydrogen bonds link the mol­ecules into layers parallel to the *bc* plane. Weak C—H⋯O hydrogen bonds and π–π inter­actions between the aromatic rings [centroid–centroid distance = 3.7674 (3) Å] further consolidate the crystal packing.

## Related literature

For applications of metal-organic frameworks with semi-rigid carb­oxy­lic acid ligands, see: Li *et al.* (2008 ▶); Chen *et al.* (2008 ▶). For background to the synthesis of various semi-rigid multicarboxyl­ate ligands, see: Maglio *et al.* (1997 ▶).
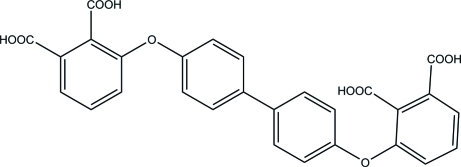


## Experimental

### 

#### Crystal data

C_28_H_18_O_10_
*M* *_r_* = 514.42
Orthorhombic, 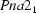

*a* = 21.5817 (7) Å
*b* = 11.2676 (4) Å
*c* = 9.5025 (3) Å
*V* = 2310.76 (13) Å^3^
*Z* = 4
Mo *K*α radiation
μ = 0.11 mm^−1^
*T* = 293 K
0.39 × 0.32 × 0.28 mm

#### Data collection

Bruker APEXII CCD area-detector diffractometer
Absorption correction: multi-scan (*SADABS*; Bruker, 2005 ▶) *T*_min_ = 0.957, *T*_max_ = 0.969
5587 measured reflections
2857 independent reflections
2352 reflections with *I* > 2σ(*I*)
*R*_int_ = 0.024

#### Refinement

*R*[*F* ^2^ > 2σ(*F* ^2^)] = 0.048
*wR*(*F* ^2^) = 0.118
*S* = 1.08
2857 reflections
343 parameters
1 restraint
H-atom parameters constrained
Δρ_max_ = 0.18 e Å^−3^
Δρ_min_ = −0.16 e Å^−3^

### 

Data collection: *APEX2* (Bruker, 2007 ▶); cell refinement: *SAINT* (Bruker, 2007 ▶); data reduction: *SAINT*; program(s) used to solve structure: *SHELXS97* (Sheldrick, 2008 ▶); program(s) used to refine structure: *SHELXL97* (Sheldrick, 2008 ▶); molecular graphics: *SHELXTL* (Sheldrick, 2008 ▶); software used to prepare material for publication: *SHELXTL*.

## Supplementary Material

Crystal structure: contains datablock(s) global, I. DOI: 10.1107/S1600536811053219/cv5208sup1.cif

Structure factors: contains datablock(s) I. DOI: 10.1107/S1600536811053219/cv5208Isup2.hkl

Supplementary material file. DOI: 10.1107/S1600536811053219/cv5208Isup3.cml

Additional supplementary materials:  crystallographic information; 3D view; checkCIF report

## Figures and Tables

**Table 1:** Hydrogen-bond geometry (Å, °)

*D*—H⋯*A*	*D*—H	H⋯*A*	*D*⋯*A*	*D*—H⋯*A*
O2—H2⋯O7^i^	0.82	1.85	2.649 (4)	164
O8—H8*A*⋯O1^ii^	0.82	1.85	2.659 (4)	169
O3—H3⋯O6^iii^	0.82	1.76	2.575 (4)	172
O5—H5⋯O4^iv^	0.82	1.92	2.732 (4)	169
C11—H11⋯O6^v^	0.93	2.46	3.284 (5)	148
C13—H13⋯O3^vi^	0.93	2.51	3.438 (6)	173
C16—H16⋯O6^v^	0.93	2.54	3.431 (6)	161

Symmetry codes: (i) 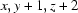; (ii) 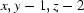; (iii) 
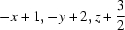
; (iv) 
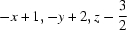
; (v) 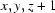; (vi) 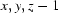.

**Fig. 1.**
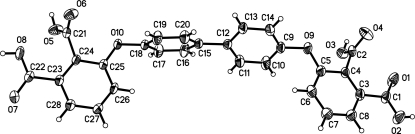
The molecular structure of (I) showing the atomic numbering and 30% probability displacement ellipsoids.

## supplementary crystallographic information

### Comment

Various semirigid multicarboxylate ligands are being used in design of metal-organic frameworks (MOFs) having various potential applications (Chen *et al.*, 2008; Li *et al.*, 2008). Herewith we report the synthesis (Maglio *et al.*, 1997) and single-crystal structure of the title compound - a new semirigid multicarboxylate ligand containing two semirigid phthalic acid groups.

In the title molecule (Fig.1), two central benzene rings form a dihedral angle of 31.0 (1)°. In the phthalic acid fragments, the carboxy groups in *meta* positions are approximately coplanar with the attached benzene rings being inclined to their planes at 2.7 (1) and 10.3 (1)°, respectively, while carboxy groups in *ortho* positions are twisted from the benzene rings at 83.5 (1) and 75.4 (1)°, respectively. In the crystal structure, intermolecular O—H···O hydrogen bonds (Table 1) link the molecules into layers parallel to *bc* plane. Weak intermolecular C—H···O hydrogen bonds (Table 1) and π–π interactions between the aromatic rings [centroid-centroid distance of 3.7674 (3) Å] consolidate further the crystal packing.

### Experimental

To a solution of 4,4'-biphenol(1.62 g,0.01 mol) and anhydrous Na2CO3(2.12 g,0.02 mol) in DMF(25 ml)stirred for 30 min, 3-nitropthalonitrile(3.46 g,0.02 mol) was added.The resulting mixture was stirred for 48 h. Then the mixture was poured into water (500 ml), and a slightly yellow solid was yielded and isolated by filtration. The crude product was dried in air, yielding 3,3'-(4,4'-biphenylenebis(oxy))diphthalonitrile. The mixture of 3,3'-(4,4'-biphenylenebis(oxy))diphthalonitrile (3.6 g, 0.01 mol) and NaOH (3.2 g, 0.08 mol) in distilled water (150 ml) was refluxed until the solution turned clear Then, the solution was cooled drown to room temperature and filtered. After the pH value of the filtrate was adjusted to about 4–5 with HCl (6.0 mol/*L*), the filtrate was kept undisturbed at room temperature. After about one day, a large amount of yellow solid of (I) was collected by filtration. A mixture containing Zn(NO_3_)_2_.6H_2_O (0.0595 g, 0.2 mmol), (I)(0.0514 g, 0.1 mmol), and H_2_O (15 ml) was sealed in a Teflon-lined stainless steel reactor and heated at 120 for 3 days. Unfortunately, X-ray quality single crystals of (I) were only obtained.

### Refinement

All hydrogen atoms were positioned geometrically and included in the refinement using a riding-model approximation [aromatic C–H = 0.93 Å, O–H = 0.82 Å] with *U*_iso_(H) = 1.2U_eq_(C, O). In the absence of any significant anomalous scatterers in the molecule, the 689 Friedel pairs were merged before the final refinement.

### Crystal data

**Table:**

C_28_H_18_O_10_	*D*_x_ = 1.479 Mg m^−^^3^
*M**_r_* = 514.42	Mo *K*α radiation, λ = 0.71073 Å
Orthorhombic, *P**n**a*2_1_	Cell parameters from 1882 reflections
*a* = 21.5817 (7) Å	θ = 3.0–29.1°
*b* = 11.2676 (4) Å	µ = 0.11 mm^−^^1^
*c* = 9.5025 (3) Å	*T* = 293 K
*V* = 2310.76 (13) Å^3^	Block, yellow
*Z* = 4	0.39 × 0.32 × 0.28 mm
*F*(000) = 1064	

### Data collection

**Table:**

Bruker APEXII CCD area-detector diffractometer	2857 independent reflections
Radiation source: fine-focus sealed tube	2352 reflections with *I* > 2σ(*I*)
graphite	*R*_int_ = 0.024
phi and ω scans	θ_max_ = 25.0°, θ_min_ = 3.0°
Absorption correction: multi-scan (*SADABS*; Bruker, 2005)	*h* = −8→25
*T*_min_ = 0.957, *T*_max_ = 0.969	*k* = −13→9
5587 measured reflections	*l* = −5→11

### Refinement

**Table:**

Refinement on *F*^2^	Primary atom site location: structure-invariant direct methods
Least-squares matrix: full	Secondary atom site location: difference Fourier map
*R*[*F*^2^ > 2σ(*F*^2^)] = 0.048	Hydrogen site location: inferred from neighbouring sites
*wR*(*F*^2^) = 0.118	H-atom parameters constrained
*S* = 1.08	*w* = 1/[σ^2^(*F*_o_^2^) + (0.0626*P*)^2^ + 0.1818*P*] where *P* = (*F*_o_^2^ + 2*F*_c_^2^)/3
2857 reflections	(Δ/σ)_max_ < 0.001
343 parameters	Δρ_max_ = 0.18 e Å^−^^3^
1 restraint	Δρ_min_ = −0.16 e Å^−^^3^

### Special details

**Table:**

Geometry. All e.s.d.'s (except the e.s.d. in the dihedral angle between two l.s. planes) are estimated using the full covariance matrix. The cell e.s.d.'s are taken into account individually in the estimation of e.s.d.'s in distances, angles and torsion angles; correlations between e.s.d.'s in cell parameters are only used when they are defined by crystal symmetry. An approximate (isotropic) treatment of cell e.s.d.'s is used for estimating e.s.d.'s involving l.s. planes.
Refinement. Refinement of *F*^2^ against ALL reflections. The weighted *R*-factor *wR* and goodness of fit *S* are based on *F*^2^, conventional *R*-factors *R* are based on *F*, with *F* set to zero for negative *F*^2^. The threshold expression of *F*^2^ > σ(*F*^2^) is used only for calculating *R*-factors(gt) *etc*. and is not relevant to the choice of reflections for refinement. *R*-factors based on *F*^2^ are statistically about twice as large as those based on *F*, and *R*- factors based on ALL data will be even larger.

### Fractional atomic coordinates and isotropic or equivalent isotropic displacement parameters (Å^2^)

**Table:**

	*x*	*y*	*z*	*U*_iso_*/*U*_eq_	
C1	0.30406 (19)	1.3782 (3)	1.6071 (4)	0.0542 (10)	
C2	0.41811 (18)	1.3386 (3)	1.4287 (4)	0.0450 (9)	
C3	0.30093 (19)	1.3259 (3)	1.4624 (4)	0.0527 (10)	
C4	0.35348 (17)	1.3116 (3)	1.3805 (4)	0.0445 (9)	
C5	0.34704 (19)	1.2629 (3)	1.2462 (4)	0.0515 (10)	
C6	0.2897 (2)	1.2302 (4)	1.1958 (5)	0.0638 (12)	
H6	0.2860	1.1980	1.1061	0.077*	
C7	0.2378 (2)	1.2454 (4)	1.2786 (5)	0.0691 (13)	
H7	0.1990	1.2236	1.2444	0.083*	
C8	0.2431 (2)	1.2924 (4)	1.4110 (5)	0.0609 (11)	
H8	0.2081	1.3020	1.4667	0.073*	
C9	0.40425 (18)	1.1761 (4)	1.0552 (4)	0.0509 (10)	
C10	0.3961 (2)	1.0575 (4)	1.0751 (4)	0.0666 (13)	
H10	0.3870	1.0279	1.1641	0.080*	
C11	0.4016 (2)	0.9812 (3)	0.9611 (5)	0.0609 (12)	
H11	0.3963	0.9001	0.9749	0.073*	
C12	0.41474 (16)	1.0225 (3)	0.8286 (4)	0.0446 (9)	
C13	0.4236 (2)	1.1432 (3)	0.8129 (5)	0.0589 (11)	
H13	0.4335	1.1738	0.7248	0.071*	
C14	0.4178 (2)	1.2195 (3)	0.9266 (5)	0.0596 (11)	
H14	0.4233	1.3007	0.9142	0.071*	
C15	0.41748 (16)	0.9389 (3)	0.7056 (4)	0.0438 (9)	
C16	0.4346 (2)	0.8221 (3)	0.7200 (4)	0.0616 (11)	
H16	0.4469	0.7950	0.8082	0.074*	
C17	0.4343 (2)	0.7437 (4)	0.6092 (5)	0.0624 (11)	
H17	0.4464	0.6653	0.6227	0.075*	
C18	0.41619 (17)	0.7812 (3)	0.4798 (4)	0.0467 (9)	
C19	0.3990 (2)	0.8959 (3)	0.4610 (5)	0.0639 (12)	
H19	0.3865	0.9217	0.3724	0.077*	
C20	0.3997 (2)	0.9744 (3)	0.5721 (4)	0.0606 (12)	
H20	0.3881	1.0529	0.5571	0.073*	
C21	0.42764 (16)	0.6151 (3)	0.1051 (4)	0.0410 (8)	
C22	0.3124 (2)	0.5020 (3)	−0.0191 (4)	0.0539 (10)	
C23	0.31384 (17)	0.5530 (3)	0.1252 (4)	0.0458 (9)	
C24	0.36704 (17)	0.6045 (3)	0.1821 (4)	0.0421 (8)	
C25	0.36412 (17)	0.6571 (3)	0.3145 (4)	0.0466 (9)	
C26	0.30915 (18)	0.6577 (4)	0.3882 (4)	0.0587 (11)	
H26	0.3074	0.6929	0.4767	0.070*	
C27	0.25716 (19)	0.6067 (4)	0.3322 (5)	0.0656 (12)	
H27	0.2202	0.6080	0.3824	0.079*	
C28	0.2594 (2)	0.5534 (4)	0.2014 (4)	0.0598 (11)	
H28	0.2241	0.5177	0.1646	0.072*	
O1	0.35545 (15)	1.4066 (3)	1.6566 (3)	0.0855 (10)	
O2	0.25406 (16)	1.3930 (3)	1.6720 (3)	0.0896 (11)	
H2	0.2612	1.4218	1.7496	0.134*	
O3	0.44618 (13)	1.2493 (2)	1.4775 (4)	0.0628 (8)	
H3	0.4811	1.2688	1.5023	0.094*	
O4	0.43977 (14)	1.4384 (2)	1.4219 (4)	0.0752 (10)	
O5	0.46202 (13)	0.5243 (2)	0.0978 (4)	0.0697 (9)	
H5	0.4936	0.5406	0.0537	0.105*	
O6	0.44146 (14)	0.7116 (2)	0.0561 (4)	0.0679 (9)	
O7	0.26478 (17)	0.4490 (3)	−0.0584 (4)	0.0890 (11)	
O8	0.35855 (15)	0.5180 (3)	−0.0969 (3)	0.0740 (9)	
H8A	0.3525	0.4863	−0.1733	0.111*	
O9	0.40081 (14)	1.2559 (3)	1.1684 (3)	0.0626 (8)	
O10	0.41860 (12)	0.7034 (2)	0.3652 (3)	0.0551 (7)	

### Atomic displacement parameters (Å^2^)

**Table:**

	*U*^11^	*U*^22^	*U*^33^	*U*^12^	*U*^13^	*U*^23^
C1	0.053 (2)	0.072 (2)	0.039 (2)	0.0091 (19)	−0.004 (2)	−0.016 (2)
C2	0.059 (2)	0.0429 (19)	0.0327 (19)	0.0044 (19)	−0.0064 (19)	−0.0080 (16)
C3	0.059 (2)	0.057 (2)	0.042 (2)	0.0023 (18)	−0.004 (2)	−0.014 (2)
C4	0.045 (2)	0.054 (2)	0.035 (2)	−0.0003 (16)	−0.0065 (18)	−0.0138 (17)
C5	0.050 (2)	0.060 (2)	0.044 (2)	−0.0032 (18)	−0.002 (2)	−0.0131 (19)
C6	0.062 (3)	0.082 (3)	0.047 (3)	−0.013 (2)	−0.013 (2)	−0.022 (2)
C7	0.052 (3)	0.098 (3)	0.057 (3)	−0.011 (2)	−0.009 (2)	−0.024 (3)
C8	0.049 (2)	0.083 (3)	0.051 (3)	−0.004 (2)	−0.001 (2)	−0.019 (2)
C9	0.054 (2)	0.060 (2)	0.039 (2)	−0.0010 (19)	−0.003 (2)	−0.016 (2)
C10	0.101 (4)	0.074 (3)	0.025 (2)	0.001 (2)	−0.008 (2)	−0.006 (2)
C11	0.082 (3)	0.054 (2)	0.047 (3)	0.000 (2)	−0.005 (2)	−0.009 (2)
C12	0.0415 (19)	0.0541 (19)	0.038 (2)	0.0004 (16)	−0.0004 (18)	−0.0093 (19)
C13	0.081 (3)	0.061 (2)	0.035 (2)	−0.013 (2)	0.005 (2)	−0.012 (2)
C14	0.076 (3)	0.053 (2)	0.050 (3)	−0.010 (2)	0.005 (2)	−0.012 (2)
C15	0.039 (2)	0.054 (2)	0.038 (2)	−0.0012 (16)	−0.0011 (18)	−0.0119 (18)
C16	0.075 (3)	0.071 (2)	0.038 (2)	0.026 (2)	−0.013 (2)	−0.009 (2)
C17	0.077 (3)	0.057 (2)	0.053 (3)	0.019 (2)	−0.001 (2)	−0.011 (2)
C18	0.044 (2)	0.053 (2)	0.043 (2)	−0.0015 (17)	0.006 (2)	−0.0169 (19)
C19	0.094 (3)	0.066 (2)	0.031 (2)	0.005 (2)	−0.008 (2)	−0.002 (2)
C20	0.089 (3)	0.049 (2)	0.044 (3)	0.003 (2)	−0.002 (2)	−0.0033 (19)
C21	0.047 (2)	0.0430 (19)	0.0329 (19)	0.0040 (16)	0.0014 (17)	−0.0074 (17)
C22	0.057 (3)	0.065 (2)	0.039 (2)	−0.001 (2)	0.010 (2)	−0.009 (2)
C23	0.051 (2)	0.0537 (19)	0.033 (2)	−0.0001 (16)	0.0028 (19)	−0.0076 (17)
C24	0.048 (2)	0.0423 (16)	0.036 (2)	0.0029 (16)	0.0074 (17)	−0.0066 (17)
C25	0.047 (2)	0.0537 (19)	0.039 (2)	−0.0015 (16)	0.0073 (19)	−0.0128 (18)
C26	0.058 (3)	0.083 (3)	0.035 (2)	−0.005 (2)	0.011 (2)	−0.024 (2)
C27	0.049 (2)	0.097 (3)	0.051 (3)	−0.012 (2)	0.016 (2)	−0.024 (2)
C28	0.054 (2)	0.081 (3)	0.045 (2)	−0.009 (2)	0.005 (2)	−0.016 (2)
O1	0.060 (2)	0.141 (3)	0.055 (2)	0.0096 (19)	−0.0108 (16)	−0.046 (2)
O2	0.070 (2)	0.148 (3)	0.0512 (19)	−0.008 (2)	0.0082 (17)	−0.042 (2)
O3	0.0580 (17)	0.0637 (16)	0.067 (2)	−0.0040 (14)	−0.0070 (15)	0.0019 (16)
O4	0.075 (2)	0.0582 (16)	0.092 (3)	−0.0139 (15)	−0.032 (2)	0.0110 (17)
O5	0.0570 (17)	0.0566 (14)	0.096 (3)	0.0102 (13)	0.0271 (18)	0.0128 (17)
O6	0.0712 (19)	0.0516 (15)	0.081 (2)	0.0075 (13)	0.0242 (17)	0.0159 (15)
O7	0.083 (2)	0.132 (3)	0.0516 (18)	−0.042 (2)	0.0072 (18)	−0.037 (2)
O8	0.067 (2)	0.111 (2)	0.0439 (18)	−0.0010 (16)	0.0045 (16)	−0.0348 (17)
O9	0.0624 (18)	0.0821 (18)	0.0433 (17)	−0.0121 (14)	0.0023 (15)	−0.0300 (14)
O10	0.0456 (14)	0.0718 (16)	0.0479 (17)	−0.0056 (12)	0.0097 (13)	−0.0281 (14)

### Geometric parameters (Å, °)

**Table:**

C1—O1	1.246 (5)	C16—C17	1.375 (6)
C1—O2	1.254 (5)	C16—H16	0.9300
C1—C3	1.497 (6)	C17—C18	1.357 (6)
C2—O4	1.219 (4)	C17—H17	0.9300
C2—O3	1.263 (4)	C18—C19	1.357 (5)
C2—C4	1.499 (5)	C18—O10	1.399 (4)
C3—C4	1.385 (5)	C19—C20	1.378 (6)
C3—C8	1.392 (5)	C19—H19	0.9300
C4—C5	1.396 (5)	C20—H20	0.9300
C5—C6	1.377 (6)	C21—O6	1.220 (4)
C5—O9	1.378 (5)	C21—O5	1.266 (4)
C6—C7	1.381 (6)	C21—C24	1.504 (5)
C6—H6	0.9300	C22—O7	1.246 (5)
C7—C8	1.370 (6)	C22—O8	1.253 (5)
C7—H7	0.9300	C22—C23	1.487 (5)
C8—H8	0.9300	C23—C28	1.380 (5)
C9—C14	1.349 (6)	C23—C24	1.395 (5)
C9—C10	1.361 (6)	C24—C25	1.392 (5)
C9—O9	1.404 (4)	C25—O10	1.373 (4)
C10—C11	1.388 (6)	C25—C26	1.378 (5)
C10—H10	0.9300	C26—C27	1.368 (6)
C11—C12	1.372 (6)	C26—H26	0.9300
C11—H11	0.9300	C27—C28	1.381 (6)
C12—C13	1.382 (5)	C27—H27	0.9300
C12—C15	1.503 (5)	C28—H28	0.9300
C13—C14	1.386 (5)	O2—H2	0.8200
C13—H13	0.9300	O3—H3	0.8200
C14—H14	0.9300	O5—H5	0.8200
C15—C16	1.374 (5)	O8—H8A	0.8200
C15—C20	1.384 (6)		
			
O1—C1—O2	123.1 (4)	C15—C16—C17	122.5 (4)
O1—C1—C3	119.2 (4)	C15—C16—H16	118.8
O2—C1—C3	117.7 (4)	C17—C16—H16	118.8
O4—C2—O3	124.7 (4)	C18—C17—C16	119.7 (4)
O4—C2—C4	121.8 (3)	C18—C17—H17	120.1
O3—C2—C4	113.4 (3)	C16—C17—H17	120.1
C4—C3—C8	120.3 (4)	C17—C18—C19	119.6 (4)
C4—C3—C1	121.7 (4)	C17—C18—O10	120.0 (3)
C8—C3—C1	118.0 (4)	C19—C18—O10	120.3 (4)
C3—C4—C5	118.6 (4)	C18—C19—C20	120.5 (4)
C3—C4—C2	124.5 (3)	C18—C19—H19	119.7
C5—C4—C2	116.8 (3)	C20—C19—H19	119.7
C6—C5—O9	123.7 (4)	C19—C20—C15	121.3 (4)
C6—C5—C4	120.8 (4)	C19—C20—H20	119.3
O9—C5—C4	115.4 (3)	C15—C20—H20	119.3
C5—C6—C7	119.9 (4)	O6—C21—O5	123.8 (3)
C5—C6—H6	120.1	O6—C21—C24	118.0 (3)
C7—C6—H6	120.1	O5—C21—C24	118.2 (3)
C8—C7—C6	120.2 (4)	O7—C22—O8	123.2 (4)
C8—C7—H7	119.9	O7—C22—C23	118.6 (4)
C6—C7—H7	119.9	O8—C22—C23	118.2 (4)
C7—C8—C3	120.2 (4)	C28—C23—C24	119.7 (3)
C7—C8—H8	119.9	C28—C23—C22	117.9 (4)
C3—C8—H8	119.9	C24—C23—C22	122.3 (3)
C14—C9—C10	120.6 (4)	C25—C24—C23	119.3 (3)
C14—C9—O9	118.3 (3)	C25—C24—C21	116.4 (3)
C10—C9—O9	121.0 (4)	C23—C24—C21	124.1 (3)
C9—C10—C11	119.3 (4)	O10—C25—C26	123.9 (3)
C9—C10—H10	120.4	O10—C25—C24	116.1 (3)
C11—C10—H10	120.4	C26—C25—C24	120.0 (3)
C12—C11—C10	121.6 (3)	C27—C26—C25	120.4 (4)
C12—C11—H11	119.2	C27—C26—H26	119.8
C10—C11—H11	119.2	C25—C26—H26	119.8
C11—C12—C13	117.5 (3)	C26—C27—C28	120.2 (4)
C11—C12—C15	120.6 (3)	C26—C27—H27	119.9
C13—C12—C15	121.8 (3)	C28—C27—H27	119.9
C12—C13—C14	120.9 (4)	C23—C28—C27	120.3 (4)
C12—C13—H13	119.6	C23—C28—H28	119.9
C14—C13—H13	119.6	C27—C28—H28	119.9
C9—C14—C13	120.1 (3)	C1—O2—H2	109.5
C9—C14—H14	120.0	C2—O3—H3	109.5
C13—C14—H14	120.0	C21—O5—H5	109.5
C16—C15—C20	116.3 (3)	C22—O8—H8A	109.5
C16—C15—C12	122.2 (3)	C5—O9—C9	119.5 (3)
C20—C15—C12	121.4 (3)	C25—O10—C18	118.6 (3)

### Hydrogen-bond geometry (Å, °)

**Table:**

*D*—H···*A*	*D*—H	H···*A*	*D*···*A*	*D*—H···*A*
O2—H2···O7^i^	0.82	1.85	2.649 (4)	164.
O8—H8A···O1^ii^	0.82	1.85	2.659 (4)	169.
O3—H3···O6^iii^	0.82	1.76	2.575 (4)	172.
O5—H5···O4^iv^	0.82	1.92	2.732 (4)	169.
C11—H11···O6^v^	0.93	2.46	3.284 (5)	148.
C13—H13···O3^vi^	0.93	2.51	3.438 (6)	173.
C16—H16···O6^v^	0.93	2.54	3.431 (6)	161.

Symmetry codes: (i) *x*, *y*+1, *z*+2; (ii) *x*, *y*−1, *z*−2; (iii) −*x*+1, −*y*+2, *z*+3/2; (iv) −*x*+1, −*y*+2, *z*−3/2; (v) *x*, *y*, *z*+1; (vi) *x*, *y*, *z*−1.
